# Response to back-to-back outbreaks of circulating vaccine-derived poliovirus type 2 in two nomadic pastoralist settlements in Oti Region, Ghana-2019

**DOI:** 10.1186/s13690-022-01021-y

**Published:** 2023-01-04

**Authors:** Donne Kofi Ameme, Yaw Ofori Yeboah, John Kofi Odoom, Senanu Kwesi Djokoto, Ernest Akyereko, Abdulaziz Mamudu, Mukaila Diwura, William Opare, Patrick Avevor, Stanley Diamenu, Sally-Ann Ohene, Ernest Kenu, Franklin Asiedu-Bekoe

**Affiliations:** 1 Ghana Field Epidemiology and Laboratory Training Programme, University of Ghana School of Public Health, Accra, Legon Ghana; 2 Public Health Division, Ghana Health Service, Accra, Ghana; 3 Volta Regional Health Directorate, Ghana Health Service, Ho, Ghana; 4 Noguchi Memorial Institute for Medical Research, Legon, Accra, Ghana; 5 Nkwanta North District Health Directorate, Ghana Health Service, Nkwanta, Ghana; 6 Krachi-Nchumuru District Health Directorate, Ghana Health Service, Krachi, Ghana; 7 Expanded Programme on Immnunization, Ghana Health Service, Accra, Ghana; 8 World Health Organization, Country Office, Accra, Ghana

**Keywords:** Poliovirus, Vaccine-derived, Circulating, Outbreak, Vaccination, Pastoralist

## Abstract

**Background:**

The global switch from trivalent oral poliovirus vaccine (OPV) to bivalent OPV in April 2016 without corresponding co-administration of inactivated poliovirus vaccine (IPV) until June 2018, created a cohort of poliovirus type 2 naïve children with risk of developing vaccine-derived poliovirus type 2 (VDPV2). In November and December 2019, two cases of circulating vaccine-derived poliovirus type 2 (cVDPV2) were confirmed in quick succession through Acute Flaccid Paralysis (AFP) surveillance in two nomadic pastoralist settlements in Oti Region. We investigated to determine the outbreak extent, identify risk factors and implement control and preventive measures.

**Methods:**

We interviewed case-patients’ families, abstracted immunization records, assessed AFP surveillance and conducted rapid OPV and IPV vaccination coverage surveys. Using AFP case definition of any child less than 15 years in the community with sudden onset of paralysis from July to November 2019 (in case-patient 1’s district) and August to December 2019 (in case-patient 2’s district), we conducted active case search. Stool samples from apparently healthy children and close contacts of the case-patients were collected and tested for poliovirus. We conducted environmental assessment of the community to identify potential risk factors.

**Results:**

Case-patient 1 was an eight-year-old female who had taken two doses of OPV while case-patient 2 was an eight-month-old male who had taken three out of required four OPV doses in addition to IPV at seven months. Families of both case-patients had either travelled to or received visitors from areas with confirmed cVDPV2. Of all children surveyed, eight (29.6%) of 27 and three (18.8%) of 16 eligible children in communities of case-patient 1 and 2 respectively had received required four doses of OPV. No AFP case was found in both communities and surrounding settlements. Both communities had no source of potable water and toilet facilities. A stool sample from a contact of case-patient 1 tested positive for cVDPV2.

**Conclusion:**

Outbreaks of cVDPV2 occurred in insanitary, under-vaccinated nomadic pastoralist settlements in Oti Region. Three rounds of monovalent OPV vaccination campaigns for children under 5 years of age in the districts and region as well as countrywide IPV vaccination campaign for poliovirus type 2 naïve cohort were conducted.

## Background

Poliomyelitis (polio), a highly contagious faecal-orally transmitted viral disease, remains a major public health concern [1]. The disease commonly affects children less than five years old and manifests as acute flaccid paralysis (AFP) capable of resulting in irreversible paralysis or death [2]. Of the three poliovirus types 1 , 2 and 3 that cause poliomyelitis, wild poliovirus type 2 was has not been detected since 1999 and was declared eradicated in 2015 while wild poliovirus type 3 was last reported in 2012 [3, 4]. This was accomplished by the wide use of live attenuated oral polio vaccine (OPV) [5]. This leaves wild poliovirus type 1 and vaccine-derived polioviruses (VDPV) as the main agents responsible for polio events with VDPV currently outnumbering the wild types [6].

Vaccine-derived poliovirus occur in settings with low immunization rates and poor sanitation when oral polio vaccine gets excreted in stool and spread from one unvaccinated child to another over a prolonged period of time. The virus can thus mutate into a form that can cause paralysis [7]. Unlike with the inactivated (killed) injectable polio vaccine, this phenomenon occurs only with the live attenuated (weakened) oral polio vaccine, that provides immunity in the gut where the polio virus replicates.

Though poliomyelitis predominantly affects infants and young children under five years of age; population groups not covered by vaccination such as nomads, refugees and internally displaced persons are at high risk [8, 9]. Polio vaccine given multiple times remain an effective public health tool for protection of children against the infection [10]. Oral polio vaccine and inactivated polio vaccine (IPV) have contributed significantly to reducing the case load of poliomyelitis. Globally, wild poliovirus cases have decreased by over 99% from an estimated 350,000 cases in more than 125 endemic countries since the Global Polio Eradication Initiative was launched in 1988 to 33 reported cases in 2018 [3]. In Ghana, a total of 54 wild polioviruses (all type 1) were recorded from 1996, when Ghana launched its polio eradication initiative, to 2008, when the last wild poliovirus was detected in the Northern region [11]. Ghana was later certified polio free in 2015 after over five years of having no reported case of poliovirus [12]. 

In the face of the eradication of poliovirus type 2, continuous administration of trivalent oral poliovirus vaccine (tOPV), which contains types 1, 2 and 3 live attenuated polioviruses posed a potential risk of paralytic poliomyelitis associated with OPV type 2 particularly in settings with poor sanitation [13]. Indeed, in 2015, there was emergence of circulating vaccine-derived poliovirus type 2 (cVDPV2) in several countries with outbreaks reported in five countries worldwide namely Burma, Guinea, Nigeria, Pakistan and South Sudan [5]. From April 2016, these developments necessitated a synchronized global switch from tOPV effective against all three strains of polioviruses; to bivalent oral poliovirus vaccine (bOPV) effective against poliovirus types 1 and 3 [5]. As part of the Global Polio Eradication Initiative Endgame Strategic Plan 2013-2018, at least one dose of inactivated poliovirus vaccine (IPV) is recommended for inclusion in childhood immunization schedule to complement the switch from tOPV to bOPV in order to further reduce the risk of type 2 poliovirus outbreaks [14]. However, in Ghana, it was not until June 2018 when IPV which was planned to commence in the fourth quarter of 2017 due to global IPV supply constraint, became available to be given to children [15, 16]. This generated a cohort of children naïve to poliovirus type 2 and therefore at risk of developing vaccine-derived poliovirus type 2 (VDPV2). From July 2019, Ghana’s decade-long polio free status was interrupted by the detection of cVDPV2 in the environment in the Northern Region and later in both humans and the environment in other regions of the country [17].

In November and December 2019, two successive cases of cVDPV2 were reported in two communities with similar socio-cultural and economic characteristics in two districts in the Oti region of Ghana. The first was on November 19, 2019 when a vaccine-derived poliovirus type 2 (VDPV2) was confirmed by Polio Laboratory of Noguchi Memorial Institute for Medical Research (NMIMR) from an AFP case detected during community-based surveillance in Abunyanya Number 2 in the Nkwanta North District in the Oti Region. The mother of the child took her to church for prayers on account of the weakness in the right leg. A Community-based Surveillance Volunteer (CBSV) who was present at the church service where the ill child was being prayed for, followed up to the house to assess the situation. He alerted the district Disease Control Officer leading to investigation of the case as AFP. Stool samples taken on October 29 and 30, 2019 and transported NMIMR on October 31, 2019 turned out positive for VDPV2. The national Disease Surveillance Department (DSD) was notified same day of laboratory confirmation and the head of DSD briefed the National Technical Coordinating Committee (NTCC), a national level multisectoral committee made up of professionals with technical expertise in management of public health emergencies. Following that, a multi-sectoral team of investigators was deployed on November 20, 2019 to support the Oti regional and Nkwanta North district teams to respond to the outbreak.

On December 12, 2019, while the first outbreak response was being concluded, DSD received another report from the Polio Laboratory at NMIMR of a confirmed case of cVDPV2. The cVDPV2 was confirmed from an AFP case reported by Krachi Nchumuru District also in the Oti Region. The AFP case was detected on November 3, 2019 by the district disease control and surveillance team during a mop up vaccination and case search exercise in the district. Two stool samples collected from the case-patient on November 6 and 7, 2019 respectively and transported to NMIMR on November 8, 2019 tested positive for VDPV2. Following this report, a team was deployed on December 14, 2019 to support the district and region to investigate and respond to the event. We investigated both events to determine the magnitude, source and risk factors as well as initiate control and preventive measures. This paper details the response strategies implemented to control the outbreak.

## Methods

### Outbreak setting

The outbreaks occurred in Nkwanta North and Krachi Nchumuru districts located in the northern part of the Oti region, which shares border with the Northern Region of Ghana. The Nkwanta North District, which shares borders with neighboring Togo has four sub-districts and 104 communities with 20 health facilities comprising: two health centres and 13 Community-based Health Planning and Services (CHPS) compounds, four clinics and one private maternity home. The district has population of about 78,180 persons with the population of children under 5 years estimated as 15, 636. The outbreak occurred in Abunyanya Number 2, a nomadic pastoralist community which is about 2km from the district capital.

**Fig. 1:**
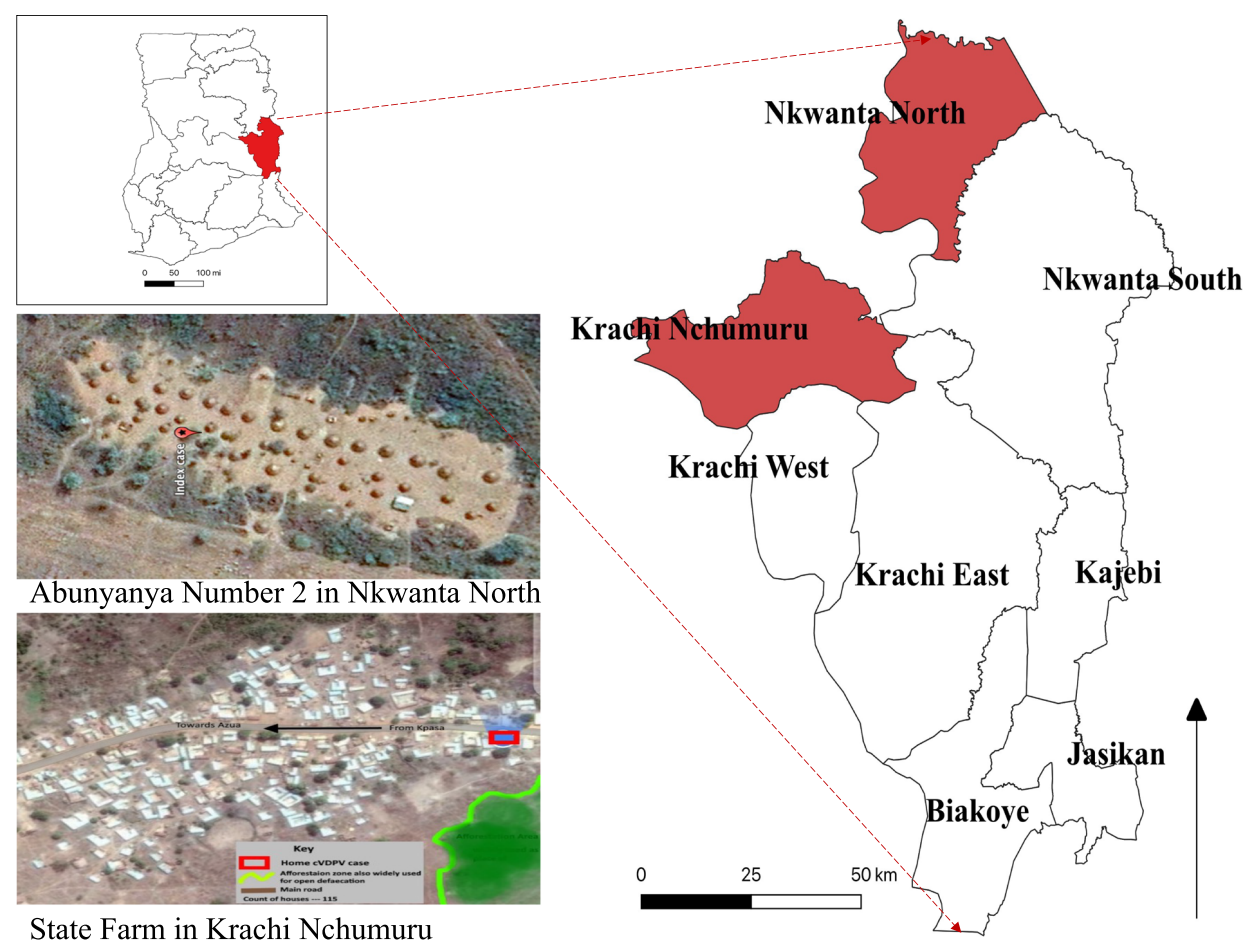
Map of the Oti region showing Nkwanta North and Krachi Nchumuru districts and Abunyanya Number 2 and State Farm communities, 2019

The Krachi Nchumuru district on the other hand shares borders with the Northern region of Ghana (Figure 1). The Northern Region had reported a series of human and environmental cases of cVDPV2 in the second half of 2019. The district has a population of 88,590 with the population of children under 15 years estimated to be 37,208. There are six sub-districts with 16 health facilities comprising: five health centres, 10 CHPS compounds and one clinic. The outbreak occurred in an isolated nomadic pastoralist settlement in State Farm, a suburb of Banda subdistrict. The nearest health facility is a CHPS compound which is about 1.5 km from the settlement. The community has about 40 housing structures most of them made of thatch. Children under five years in the community were about 20 in number. Members of the community had close ties with the Northern region of Ghana where they visit and also seek health care. Some also travel to and receive visitors from neighbouring West African countries including Nigeria which had reported cases of cVDPV2. The sub-district has a daily market which serves residents from other sub-districts in the district.

### Outbreak investigation and response design

The outbreak was planned and responded to by the practical application of the WHO standard operating procedures for responding to poliovirus event or outbreak [18]. It took into consideration key response strategies and the general principles of outbreak investigation and response.

### Outbreak response strategies

Response sub-teams were formed namely case-management, surveillance, laboratory, vaccination as well as risk communication and social mobilization. Each sub-team had a lead person who coordinated the team’s activities. The sub-teams were assigned specific tasks in line with the response strategies (Table 1). The response team met daily where every sub-team under their respective leaderships provided feedback on their activities, which were harmonized and the way forward charted.

**Table 1:** Outbreak response sub-teams and their activities, Krachi Nchumuru and Nkwanta Districts, 2019

**Sub-Team**	**Activities**
Case-management	●Engaged the case-patients’ families ●Interviewed families of case-patients ●Conducted clinical assessment of case-patients ●Conducted environmental assessment
Surveillance	●Conducted active case-finding ●Conducted rapid assessment of the AFP surveillance system ●Conducted environmental assessment
Laboratory	●Collected, packaged and transported stool sample from contacts of case-patients and health children in the communities ●Tested stool samples collected ●Conducted environmental assessment
Vaccination	●Conducted vaccination coverage survey ●Assessed the immunization system ●Conducted environmental assessment
Risk communication and social mobilization	●Conducted environmental assessment ●Engaged community members

### Interview and clinical assessment of case-patients

The investigation team went to the communities to interview the families of the case-patients. We visited the case-patients and took detailed history of the children’s illness from their respective parents and other caregivers. The children were examined to assess their general condition, muscle tone, power and residual paralysis in the affected limbs.

### Active case-finding, close contact sampling and targeted healthy children stool sampling

Sample size was not determined as all children under 15 were included in the active case finding and all children less than five years and their parents or legal guardians were included in the vaccination coverage assessment. Close contact sampling and healthy children stool sampling were based on standard sample sizes of 3 and 20 respectively [18]. The records reviewed at the health facilities covered the period July to November 2019.

Children less than 15 years were included when their parents gave consent on their behalf whilst adults above 18 years who were available, willing and gave consent were included in the vaccination coverage and surveillance assessments.

We defined a case of AFP as any child aged less than 15 years with acute paralysis of any part of the body [19] with onset from July to November 2019 in Nkwanta North district and August to December 2019 in Krachi Nchumuru district. Using the case definition, we conducted active case search for AFP cases in 279 households in Abunyanya Number 2 and adjoining communities and all 50 households in State Farm. It is recommended that at least 200 households are visited for case search in a community with a confirmed poliovirus disease with the proviso that the number of houses to be visited should take into consideration population density and other risk factors [18]. We met this target in Abunyanya Number 2 community and visited all 50 existing households in State Farm. The households were selected for visit by using random walk sampling approach within the community [20] and based on information obtained from the community members. Other places visited for case-finding were healing centres and health facilities. All the healing centres and health facilities within the catchment area of the index cases were visited for active case-finding. At the health facilities, we conducted retrospective case-finding by reviewing health facility records for missed cases of AFP.

Sample size, eligibility criteria and sampling technique for the targeted healthy children stool sampling and close contact sampling were based on the World Health Organization (WHO) recommendations [18]. The recommended sample size and eligibility for targeted healthy children stool sampling is 20 children aged less than five years old but preferably less than two years old. We conducted targeted healthy children stool sampling to determine the geographic extent of spread of the outbreak. We visited households within the communities of the index cases. The households within the community of each AFP case-patient were selected using the random walk sampling technique. In each household, all apparently healthy children (children without AFP and not a close contact with AFP case-patient) less than five years were selected for the stool sampling. We collected stool samples from 20 and 15 apparently healthy children aged less than five years for case patient 1 and 2 respectively.

Stool sample from three close contacts of case-patient 1 and two close contacts of case-patient 2 had been collected for laboratory confirmation of poliovirus in accordance with WHO guidelines, as the stool samples collected from the case-patients were inadequate. Priority for selection for close contact sampling was given to children in frequent, direct contact with the AFP cases including siblings and other household playmates younger than five years of age [21]. The World Health Organization standard operating procedure for responding to poliovirus events or outbreaks stipulates collection of stool sample from three close contacts to support the diagnosis of poliovirus in instances where the stool sample from the AFP case-patient is inadequate. The guideline also recommends targeted stool survey, where stool samples are collected from 20 apparently healthy children in the community of the cases for laboratory confirmation in order to determine the geographic extent of transmission of the infection. [18]. In our case, we collected stool sample from two close contacts of case-patient 2 because only two children aged less than five years who qualified as close contacts of the case-patient were available. Also, for the targeted healthy children stool sampling for case-patient 2, only 15 eligible children were able to produce stool samples. Stool samples were transported on reverse cold chain to the polio laboratory at NMIMR for testing.

### Assessment of immunization system and AFP surveillance

We assessed the immunization system of the districts by reviewing administrative records for routine OPV and IPV vaccination coverages as well as vaccination coverages for supplementary immunization activities (SIA) as well as key AFP surveillance indicators. We assessed OPV and IPV vaccination status of all children aged 0-59 months in the affected communities. In addition, we assessed uptake of vaccines during SIAs as well as vaccination card retention. These assessments were done by visiting households and sampling children under five years for assessment. The households were sampled using the random walk sampling technique. In each household visited, we randomly selected one child less than five years and reviewed the Child Health Record Book (CHRB), which contained the child’s vaccination records. We also interviewed the parents or guardians of the children for their vaccination history especially in instances where the CHRBs were not available.

We also assessed the immunization cold chain in the districts and facilities where community members accessed healthcare.

Rapid assessment of AFP surveillance in the district was conducted by interviewing all available surveillance focal persons at the district level and health facilities in the catchment area of the case-patients. We obtained data on the surveillance focal persons’ knowledge on AFP surveillance.

### Environmental assessment, community mobilization and health education

The team further assessed the community’s environment for cleanliness, source of water and availability of toilet facilities. Whilst assessing the environment, we educated the community members on poliomyelitis, personal and environmental hygiene.

### Data analysis

Data obtained from the response was entered into Microsoft Excel and cleaned prior to analysis. Descriptive data analysis was carried out where ages were expressed as ranges. Categorical variables were expressed as frequencies and relative frequencies. Proportion of children who received a particular vaccine dose was calculated by expressing the number of children with a particular vaccine dose as a percentage of the total number of children assessed. The knowledge of surveillance officers was scored as adequate if they knew the case definition for AFP, at least three differential diagnoses of AFP and all elements of stool adequacy (which is two stool specimens at least 8 grams, collected within 14 days of paralysis onset, collected at least 24 hours apart, received in the laboratory in good condition below ie. temperature of 8 °C, without leakage or desiccation and with proper documentation). Otherwise, knowledge was deemed inadequate. Secondary data from immunization activities were collected and assessed. Key indicators of OPV1, OPV3, IPV and dropout rates were calculated for the respective years. The vaccination coverages for the SIAs were also collected and assessed.

## Results

### Description of case-patients

**Case-patient** 1, an eight-year-old female, lived with mother in the Northern region until when she was about a year old when the mother came to Abunyanya Number 2 in the Oti region. Both parents are farmers who have had no formal education.

The case-patient often travels with the parents to a farm settlement close to the Northern Region where they usually spend some days on the farm. It was from one of such trips from the farm on October 14, 2019 when she complained of pain in the right leg, and subsequent difficulty in walking associated with fever. She was unable to lift or walk on the right leg for three days after which she gradually started limping on the right leg. There was no history of trauma.

After delivery, the index case made her first contact with the health facility at the age of six weeks where she received her first dose of vaccinations. The vaccination record from her CHRB showed that she had received two doses of tOPV from the Northern Region. (Table 2)

**Table 2:** Polio vaccines received by the index cases of circulating vaccine-derived poliovirus type 2, Krachi Nchumuru and Nkwanta Districts, 2019

**Vaccine**	**Recommended Date**	**Date Given**	
		**Case-patient one**	**Case-patient two**
**OPV**			
At Birth	At Birth	Not received	Not received
1st	6 weeks	6 weeks^a^	13 weeks^b^
2nd	10 weeks	29 weeks^a^	25 weeks^b^
3rd	14 weeks	Nil	35 weeks^b^
**IPV**			
IPV	14 weeks	*	35 weeks

^a^tOPV

^b^bOPV

^*^ IPV not introduced into routine childhood immunization at that time

**Case-patient** 2 was an eight-month-old boy living with herdsmen parents in a nomadic pastoralist settlement in Krachi Nchumuru District. He was born at home and made his first contact with a health facility at the age of three months when his parents travelled to the Northern Region to seek health care for him on account of fever. He received his first dose of vaccinations comprising OPV1 on his visit to the health facility. On September 27, 2019, two days after receiving his second dose of vaccinations including OPV2 and other injectable vaccines given during a vaccination outreach to the settlement, the parents noticed weakness in his right leg and later all four limbs. There was no prior history of trauma. With the exception of the travel with parents to the Northern Region for seeking health care, the child had never travelled out of the district. The family however received visitors from Nigeria and communities in the Northern region during a funeral in the community a month prior to the paralysis. In all, he received 3 doses of bOPV and one dose of IPV (Table 2). The two doses of OPV were received prior to onset of paralysis.

On examination, case-patient 1 looked well and walked without any pains but with a subtle abnormal gait. Muscle bulk, power and tone in all limbs were normal. For index case-patient 2, a focused neurologic examination revealed reduced muscle bulk in the right lower limb. The right leg was paralysed with significantly reduced muscle tone in the right lower extremity. There was no rigidity bilaterally. Examination of the right lower limb revealed 0/5 motor power of hip flexion and extension, 1/5 power of knee flexion and extension, and 1/5 power of toe plantar flexion and extension. Left lower limb had power of 3/5 power of hip flexion and extension, 3/5 power of knee flexion and extension, as well as 5/5 power of toe plantar flexion and extension. Both upper extremities exhibited full power throughout examination. Sensation to pain was intact on both lower limbs. Babinski and patellar reflexes were mute bilaterally.

### Active case-finding and vaccination coverage survey

Of the 279 households visited in Abunyanya Number 2 and all 50 households visited in State Farm, no case of AFP was found. The ages of the 32 children in Abunyanya Number 2 and 20 children in State Farm whose OPV and IPV vaccination status were assessed ranged from three weeks to 51 months and 26 days to 59 months respectively. Thirty (93.7%) out of the 32 children in Abunyanya Number 2 had their CHRBs available while 16 (80.0%) of the 20 children in State farm had CHRBs. Of the 32 children, 27 (84.4%) were expected to have received 4 doses of OPV vaccine per their age. However, only 8 (29.6%) out of the 27 had received all 4 doses. Similarly, in State Farm, out of 16 eligible children expected to have received 4 doses of OPV vaccine per their ages, only 3 (18.8%) had received all. All the children included in the rapid vaccination coverage survey except for two in State Farm were born after the last SIAs held in Nkwanta North and Krachi Nchumuru districts in 2014 and 2015 respectively.

### AFP surveillance assessment

At all five health facilities visited within the catchment area of case-patient 1, there was at least one surveillance focal person per facility. Patients’ records were also readily available with diagnoses stated. All focal persons had received some training on surveillance within 6 months prior to the assessment. However, there was no evidence of record review by surveillance focal persons in all five health facilities visited. Three out of five health facilities have reported at least a case of AFP within six months prior to the assessment. There were no Integrated Disease Surveillance and Response (IDSR) Technical Guideline booklets available in all the facilities visited and no evidence of findings or recommendations from supervisory visits. Knowledge of focal persons in the health facilities on AFP surveillance was inadequate.

Within the catchment area of case-patient 2, all the health facilities visited, except one, had focal persons for surveillance. None of the focal persons had received formal training on surveillance within 6 months prior to assessment. Though, there were documents available at the district health office suggesting records were reviewed at the health facilities, there was no evidence of record review in the health facility registers. There were no IDSR Technical Guideline booklets available in all the facilities visited even though two of the booklets were available at the district health office. With the exception of one CHPS compound, no evidence of supervisory visits was observed in all the other facilities. The district Disease Control Officer had adequate knowledge on AFP surveillance. However, the focal persons in the health facilities had inadequate knowledge on AFP surveillance particularly case definition and differential diagnosis of AFP. They, however understood and described in detail stool collection procedure for AFP cases. In both districts, all the reporting sites were prioritized as low, medium and high for regular active surveillance visits. Frequency of visits were accordingly assigned to the reporting sites as recommended [22].

Non-polio AFP rate for both districts have been above the target of 2 per 100, 000 population aged less than 15 years per year for the three years preceding 2019 [23]. Stool adequacy also was recorded as 100% for all the three years (2016-2018) and the year of the outbreak (Table 3).

**Table 3:** Acute Flacid Paralysis surveillance performance indicators, Nkwanta North and Krachi Nchumuru Districts, 2016-2019

**Year**	**Nkwanta North**		**Krachi Nchumuru**	
	**Non-polio AFP Rate/100, 000**	**Stool Adequacy (%)**	**Non-polio AFP Rate/100, 000**	**Stool Adequacy (%)**
2016	6.5	100	2.9	100
2017	6.4	100	11.2	100
2018	9.1	100	5.5	100
2019	6.7^a^	100	2.7^a^	100

^a^Annualized non-polio AFP rate

### Immunization system assessment

The OPV and IPV vaccination coverages for 2015-2019 were above 80% every year in both districts with dropout rates above 10% in 2015 and 2016 for Nkwanta North district (Tables 4 and 5).

**Table 4:** Oral Polio Vaccine and Inactivated Polio Vaccine immunization coverage, Nkwanta North District, 2015-2019

**Antigen**	**Year**				
	**2015**	**2016**	**2017**	**2018**	**2019**^**a**^
Target	2921	2919	2988	3057	3127
OPV1	4517	4485	4451	4507	3958
OPV3	3735	3782	4124	4247	3933
IPV	**	**	**	2093	3894
OPV 1 (%)	154	153.6	149	147.4	126.6
OPV 3 (%)	128	119.5	138	138.9	125.6
OPV1/3DOR (%)	17.2	15.7	7.3	5.7	0.6
IPV (%)	IPV introduced in June 2018			68.5	124.6

^a^2019 data is from Jan-October

^**^IPV not introduced into routine childhood immunization

**Table 5:** Oral Polio Vaccine and Inactivated Polio Vaccine immunization coverage, Krachi Nchumuru District, 2015-2019

**Antigen**	**Year**					
	**2015**	**2016**		**2017**	**2018**	**2019**^**a**^
Target	3290	3372		3386	3386	3544
OPV1	2944	2777		3085	3505	2636
OPV3	2792	2742		3251	3617	2793
IPV	***		***	***	2094	2803
OPV 1 (%)	89.5	82.4		110	104	74.4
OPV 3 (%)	84.9	81.3		104	107	78.8
OPV1/3DOR (%)	5.16	4.50		-5.38	-3.20	-6.07
IPV (%)	IPV introduced in June 2018				62	79.1

^a^2019 data is from January-October

^***^IPV not introduced into routine childhood immunization

For both districts, micro plans and activity reports for SIAs for 2013-2015 were available with vaccination coverages for each round of SIA. The SIA vaccination coverages for the 2013-2015 period were above 90% for all the years for both districts (Table 6).

**Table 6:** Trivalent Oral Polio Vaccination coverage for National Immunization Day Campaigns, Krachi Nchumuru and Nkwanta North Districts, 2013-2015

Year	Date	Nkwanta North			Krachi Nchumuru		
		Target	Number Vaccinated	Coverage (%)	Target	Number Vaccinated	Coverage (%)
2013	6-8 June	23785	23835	100.2	13271	12951	97.6
2013	24-26 October	23785	26 831	112.8	13271	13102	98.7
2014	18-20 October	27131	31238	115.1	15769	16222	102.9
2014	30 October-01November	27131	30645	113.0	15769	16044	101.7
2015	22-24 October	**	**	**	15935	14931	93.7

^**^District not included in the SIA

A well-functioning standard vaccine fridge was available in the cold store at both districts. Unlike Krachi Nchumuru, Nkwanta North District had a standby generator for the cold store. In both districts, temperature readings from January to December 2019 were within the recommended range of 2-8°C. The fridges were functioning and monitored twice daily as required. Sampled OPV had their vaccine vial monitor in stage 2 with majority being in stage 1.

### District emergency preparedness

Both districts had registers of members of their Public Health Emergency Rapid Response Teams (PHERRTs) with their telephone contacts, however there was no evidence of the PHERRT activities. The Krachi Nchumuru district Public Health Emergency Management Committee (PHEMC) had not met in 2019 but that of Nkwanta North district had one meeting prior to the cVDPV2 outbreak. Both districts had epidemic preparedness plans as well as copies of completed and blank case-based forms for Adverse Events Following Immunization (AEFI) as evidence of AEFI surveillance.

District Health Management Teams in both districts held weekly and quarterly meetings and minutes of their last meetings held prior to the outbreak were available. Nkwanta North district had records of cross border activities with their Togolese counterparts and had shared information on the outbreak via a common WhatsApp platform it shared with the adjoining district in Togo.

### Laboratory result

Even though sequencing of the sample from both case-patients revealed 28 nucleotide differences compared to the reference Sabin 2, only sample from case-patient 1 had environmental sample detected in Accra, Ghana as its closest sequencing match. Stool sample of one of the contacts of case-patient 1 tested positive for poliovirus. Sequencing of the sample revealed 29 nucleotide differences compared to the reference Sabin 2. None of the stool samples collected from the 15 healthy children and close contacts of case-patient 2 was positive for poliovirus.

### Environmental assessment

There was no toilet facility in State Farm while in Abunyanya Number 2, except for a handful of houses with toilet facility, majority of the community members practiced open defecation. The only source of water in each of the communities was a stream. No refuse disposal site was available in both communities.

### Public health actions taken

Adjoining districts were alerted of the outbreak and all health workers in these districts sensitized on AFP surveillance. The members of the two communities where the outbreaks were detected were educated on personal and environmental hygiene as well as importance of childhood vaccination. We also engaged the political leadership of the two districts and advocated for improved sanitation in the affected and other similar settlements. The case-patients were linked to physiotherapy care for rehabilitation.

### Coordination of response

The outbreak response coordination and resource mobilization were by the NTCC at the national level with local PHEMC at the district and regional levels being key players. The NTCC chaired by the Director General of the Ghana Health Service consisted of experts from many organizations including human, animal and environmental health, security services, NGOs and international partners including WHO, United States Centers for Disease Control and Prevention and United Nation Children’s Fund (UNICEF); which provided both technical and logistic support. Implementation of the response strategies hinged heavily on established and strong IDSR structures at all levels of health care delivery. Specific response tasks were performed at the district and community levels. Reports were generated from the district level by the multi-stakeholder investigation team and sent to the DSD at the national level. Updates were presented at the NTCC meetings for decision-making.

### Lessons learnt

The response was conducted using WHO standard operating procedures for responding to poliovirus event or outbreak as a guide [18]. Use of the standard operating procedures facilitated the investigation and response. The strong multisectoral collaboration which leveraged on existing partnerships coupled; with extensive community engagement were key success factors. Rapid mobilization of resources and deployment of the needed human resource ensured a timely response. Task sharing by formation of different sub-teams with their respective leads helped in coordination of fieldwork. The capacity built by the investigation team during the response to the first event led to improvement in response activities for the second event underscoring the need for continuous capacity development as a preparatory measure for public health emergencies. The findings from this response underscore the importance of strong IDSR strategy with emphasis on community-based surveillance as key in early detection and response to priority public health conditions.

## Discussion

Though poliovirus disease is not alien to Ghana, the country had not reported any poliovirus event for over a decade prior to the cVDPV2 outbreaks which started in July 2019. The last cases of wild poliovirus reported in the country were imported cases in 2003 and 2008 with the last indigenous case occurring in 1999 [11, 24]. Since July 2019 when the re-emergence of poliovirus in the form of cVDPV2 in the country was first detected in an environmental sample, sporadic poliovirus events in both the environment and humans had been reported from different parts of the country, lending some credence to human-to-human transmission of the virus in country. In this outbreak, the stool sample of one of the contacts of case-patient 1 was positive for cVDPV2; which also supports possible human-to-human transmission in the community.

In both outbreaks, cases were detected and reported to the district health authorities by community-based and active surveillance efforts. This highlights well established IDSR structures and strong linkages between the communities and the health sector. The missing link relates to how active the PHRRTs are at the district level. While PHERRTs at various levels of the health sector was touted as key in controlling similar outbreak in Ethiopia [25], in the current outbreak response, the PHERRT in one of the districts appeared inactive at the time of the outbreak. Also, the capacity of the PHERRT to respond to the outbreaks was limited as this was the first time the region was recording such outbreaks and the last time Ghana as a country recorded a case of poliomyelitis prior to the emergence of the cVDPVs was over a decade ago. That notwithstanding, surveillance for AFP seems to be strong in both districts. The key AFP surveillance indicator of non-polio AFP rate for the two districts surpassing WHO recommended target of 2 per 100, 000 population aged less than 15 years for all four years assessed signifies a very sensitive AFP surveillance. Similarly, the proportion of AFP cases with adequate stool samples for the two districts which had been above recommended targets of 80% for same years demonstrates high-quality AFP surveillance in the districts [23, 26]. Except for the inadequate knowledge of health facility surveillance focal persons on AFP surveillance, the performance of AFP surveillance in both districts was generally very sensitive and of high quality.

The cohort of children susceptible for poliovirus type 2 infection in Ghana include children born from January 2016 to February 2018. Even though both case-patients were not part of this cohort, the delay in receipt of vaccinations in addition to the pertaining unhygienic conditions in their communities were risk factors for development of the disease. Evidence suggests that children living in settings with poor sanitation and low vaccination coverages are more susceptible to cVDPV [27–29] . Even though administrative data indicate very high routine OPV and IPV vaccination coverages in both districts, it is likely that certain pockets of special populations which are highly mobile including these nomadic pastoralist settlements may not have been adequately served for various reasons. Globally, migrant populations including nomads are one of the communities that are mostly underserved with health and social services [9]. It was therefore not surprising that the rapid vaccination coverage survey conducted in these communities yielded very low coverage rates. In addition, the communities were underserved regarding provision of potable water, toilet and refuse disposal facilities. These conditions disproportionately increase the risk of poliovirus outbreaks. Disease outbreaks among nomads are not rare, because their continual mobility restricts access to health services, particularly where extended course of interventions such as childhood immunization are required [30]. This was observed in a cVDPV2 outbreak in pastoralist community in Ethiopia where there was a large number of children who had never been vaccinated [25]. This underscores innovative approaches to reaching these children with vaccination and other health services. In the current outbreaks, health authorities in the two districts had identified the pastoralist communities and devised strategies for reaching them with health services. These strategies included use of mobile vaccination teams and recruitment of CBSVs from among them to report unusual health events and mobilize them for health services. However, because of their nomadic patterns coupled with some cultural beliefs they are unable to access the vaccines at all or access them in a timely manner as evidenced by the vaccination history of the case-patients and results of the rapid vaccination coverage surveys. Delivery of joint veterinary, childhood vaccination and women services [31] could therefore be useful in these settings as these combined services present opportunity for the nomads to get health services for their animals as well as allow the health workers to reach them, and their children. Despite the challenges of access to vaccination services, the district routine vaccination coverages exceeded 100% in some instances purely because of challenges with the determination of target populations which are projected from national representative surveys. This is not uncommon partly also because of the distribution of health facilities. Some children especially those close to district boundaries and national borders access vaccination services from health facilities, which are closer to them in an adjoining district than the nearest health facility in their district or country of origin.

Both case-patients had travelled to the Northern region prior to development of paralysis. It is unclear whether they contracted the disease from there. However, what is noteworthy is the fact that the Northern region of Ghana has been notorious for poliovirus outbreaks. In fact, the last indigenous and imported cases of wild poliovirus and the first cases of this new wave of cVDPV2 outbreaks in Ghana, were all recorded in the Northern region [11]. The first ever case of cVDPV2 both from the environmental and human samples were all from the Northern region [17]. It is therefore not far-fetched that the cVDPV2 could have linkages with what was occurring in the region. In both cases, the communities interacted closely with nationals from Togo and Nigeria where cVDPV2 had earlier been reported [32]. In the case of case-patient 1, the community had cross border interactions with residents of Togo while case-patient 2 had family members and other community members travelling to and from Nigeria. Possibility of introducing the infection into the community from these countries could therefore not be ruled out as concurrent cVDPV outbreaks have been reported in countries with continuous population subgroup movement between them [33].

We also considered delay in receipt of vaccination as a major risk factor for development of the cVDPV2 in both case patients. Though at the individual level, this may not be problematic especially if that vaccine is eventually received, delay in receipt of vaccines at the population level affects the fraction of vaccinated population necessary for disease elimination [34] and builds a pool of susceptible individuals which may predispose to outbreaks [35]. Beyond this, case-patient 1 had incomplete vaccination for age and therefore unlikely to benefit from the protective effect of multiple doses of OPV.

The rapid occurrence of the outbreaks in different districts in the same region, though challenging, had some silver lining. It provided opportunity for capacity building for responding to polio outbreaks in the affected districts and the region. The regional health officials who were supported from the national level in the response to case-patient 1 took up more leadership roles in the second outbreak as a result of enhanced competencies.

Though the outbreak response was rigorous, it had some shortcomings worth considering. Even though they tested positive for poliovirus, both case-patients were detected, and their stool samples collected after 14 days of illness onset. This rendered the stool samples inadequate. However, close contact sampling was implemented as a measure to support the diagnosis

Another limitation is the time used to respond to both outbreaks exceeded the stipulated 48 hours required for completion of polio case investigation. The regional and national teams took a while in joining the district teams to respond. This is because of the distance of the outbreak location from the regional and national capitals where investigation teams were mobilized to support the district capacity to investigate. The road networks to the affected districts were bad making accessing the outbreak settings difficult. Another factor that contributed to the delay in completing the investigation was the fact that the teams from the region and national level had to cross the Oti River via a ferry, which was only available based on human and vehicular traffic.

## Conclusion

Back-to-back outbreak of cVDPV2 which occurred in nomadic pastoralist settlements in the Nkwanta North and Krachi Nchumuru Districts were limited to two case-patients. The outbreaks were not found to be epidemiologically linked. Though the source of the outbreaks were not identified, there was a high probability of introduction of the poliovirus in both communities from either the Northern region or from neighbouring countries where cases of cVDVP2 had earlier been detected.

The most likely risk factors for the outbreak were delay in immunization of the child, low immunization coverage of the community, frequent interaction of community with settings with cVDPV2 outbreak. Lack of potable water, toilet facilities and practice of open defaecation were probable contributing factors.Immediate mop up vaccination exercises were conducted for children in the affected communities.

Reactive vaccination campaigns with monovalent OPV were immediately conducted in the districts and the whole region to halt transmission. A countrywide IPV vaccination campaign was later conducted for the poliovirus type 2 naïve cohort. Boreholes have been dug for Abunyanya Number 2 community whilst State Farm has been provided with makeshift toilet facilities. Surveillance has been enhanced in the affected settlements.

## Data Availability

All data generated or analysed during this study are included in this published article

## Abbreviations

AEFI: Adverse Events Following Immunization
AFP: Acute Flaccid Paralysis
CBSV: Community-based Surveillance Volunteers
CHPS: Community-based Health Planning and Services
CHRB: Child Health Record Booklet
cVDPV2: Circulating Vaccine-Derived Polio Virus Type 2
DSD: Disease Surveillance Department
OPV: Oral Polio Vaccine
IDSR: Integrated Disease Surveillance and Response
IPV: Inactivated Polio Vaccine
NMIMR: Noguchi Memorial Institute for Medical Research
NTCC: National Technical Coordinating Committee
PHEMC: Public Health Emergency Management Committee
PHERRT: Public Health Emergency Rapid Response Team
WHO: World Health Organization
VDPV: Vaccine-Derived Poliovirus
